# Impact of Health Education on Infectious Disease Knowledge in Indigenous Communities in Northwestern Malaysia

**DOI:** 10.3390/tropicalmed10070191

**Published:** 2025-07-09

**Authors:** Barathan Muttiah, Wathiqah Wahid, Alfizah Hanafiah

**Affiliations:** 1 Department of Medical Microbiology and Immunology, Faculty of Medicine, Universiti Kebangsaan Malaysia, Cheras, Kuala Lumpur 56000, Malaysia; barathanmuttiah@ukm.edu.my (B.M.); wathiqah.wahid@hctm.ukm.edu.my (W.W.); 2 Department of Parasitology and Medical Entomology, Faculty of Medicine, Universiti Kebangsaan Malaysia, Cheras, Kuala Lumpur 56000, Malaysia; 3 Infectious Diseases & Entomology Research League (IDEAL), Faculty of Medicine, Universiti Kebangsaan Malaysia, Cheras, Kuala Lumpur 56000, Malaysia

**Keywords:** health literacy, infectious diseases, community-based health education, indigenous health, Semai, Malaysia, hygiene practices, knowledge intervention

## Abstract

Indigenous people possess unique health literacy issues and challenges with preventing infectious diseases. This research assessed the baseline knowledge and misinformation in the Semai indigenous subgroup in Perak state, Malaysia, and the impact of a culturally adapted health education intervention. A single-group pre-test/post-test design was used with 156 participants ranging from 7 to 69 years old, predominantly children. The survey addressed key issues of head lice, intestinal parasites, tuberculosis (TB), handwashing, and germ transmission. An interactive, multi-station health education session in the local language produced a significant increase in overall knowledge (mean score increased from 3.17 to 3.83 out of 5, *p* < 0.0001), with the largest increase among the adult group aged 31–50 years. This was most notable for handwashing knowledge, which had the greatest increase, and misconceptions about intestinal worms and head lice remained. Differences in outcome by age suggest the need for targeted educational strategies, particularly for teenagers and elderly individuals who achieved less gain. The results support the effectiveness of culturally tailored, community-based health education in promoting the awareness of disease among indigenous communities. The drawbacks are convenience sampling, the child dominance of the sample, and the short-term follow-up. Future emphasis should be placed on long-term, community-based intervention using culturally tailored content and digital media.

## 1. Introduction

Hygiene, the prevention of infectious diseases, and health education are the mainstays of public health. They allow people to prevent disease, access early medical attention, and lead healthier lives, thus reducing the burden of disease, improving the well-being of communities, and alleviating the burden on the health system [1]. In Malaysia, the native Orang Asli of Peninsular Malaysia, numbering around 149,500 or 0.7% of the population [2], possess traditional ecological knowledge and customary practices implemented to protect natural resources and lower disease risk [3]. A few of these include the utilization of medicinal herbs such as Tongkat Ali and Kacip Fatimah, the rotation of crops, preservation of sacred groves, and adherence to customary rituals for hygiene and quarantine protocols [3,4]. These beliefs have solid roots in indigenous cosmology and are often followed at the cost of Western medicine, which is often considered a last option [4].

Despite their cultural health, there is extensive knowledge shortfall and misinformation regarding disease causes among Orang Asli people, especially when access to medical care is limited. For example, illnesses such as tuberculosis or malaria may be attributed to spiritual punishment rather than microbial origin [5], and measles vaccines have been refused due to misinformation, and vector control has been ineffective due to the low literacy about dengue transmission [6,7]. Misconceptions thus undermine health-seeking behavior, slow treatment, and discourage public health interventions. Although earlier studies have considered Orang Asli health in general [3,4,5,6,7,8,9,10,11,12,13], only a few have investigated the level of health literacy, food security along with nutrition, habits of hygiene, risk of infectious diseases like scrub typhus, and deep-rooted misconceptions among the Semai subgroup.

Additionally, the relationship between traditional medicine and modern medicine is complex. There is indigenous medical pluralism, where spiritual ailments are treated by traditional healers and emergencies are treated at modern clinics [2]. For instance, Aboriginal Australians mix bush medicine and biomedical treatments [14]. Suspicion of modern health care is an obstacle unless adapted to the local culture. Conversely, providers of modern medicine may denigrate local knowledge, creating suspicion and limited cooperation. Models that respect both systems have been effective in promoting improved health outcomes [15].

This study was prompted by the demand to measure health literacy and overall myths concerning infectious diseases among the Semai community of Perak, Malaysia. Specifically, this study sought to conduct baseline endemic infectious disease knowledge. This study also measured the effectiveness of a culturally adapted community health education intervention in facilitating knowledge and awareness. Because it is a case study of a single subgroup of indigenous people in one region, the findings are to be interpreted within the Semai subgroup and cannot be extrapolated across a broader population. By identifying and filling specific knowledge gaps and myths, the findings are able to inform the provision of insights into the design of future targeted and culturally relevant health education programs. Lastly, this study adds to larger efforts to reduce health inequities in indigenous peoples and promotes equitable public health outcomes.

Conceptually, this study is based on the Health Belief Model (HBM), in that it assumes that individuals are more likely to adopt health-protective habits as long as they perceive themselves to be at risk for a health threat, categorize the threat as serious, assume that action would reduce the risk of the disease, and perceive few obstacles to action. By augmenting information on infectious disease transmission and prevention, the intervention was designed to influence primary HBM constructs such as perceived susceptibility, perceived severity, and perceived benefits. Use of education that is culturally sensitive and community-oriented is designed to increase self-efficacy and lower perceived barriers, furthering extended health behavior change.

## 2. Methods

### 2.1. Study Design and Sample

A single-group pre-test/post-test design was conducted in November 2024 among the Semai, one of the subtribes in the indigenous community in Perak, Malaysia. Participants were recruited through convenience sampling from individuals attending a community-based health education program. Participation was voluntary and informed consent was obtained before enrolment. For participants under 18 years old, consent was provided by a parent or guardian.

The inclusion criteria were as follows:

- Identified as Semai ethnicity;
- Aged 7 years and above;
- Able to provide informed consent.

The exclusion criteria were as follows:

- They did not complete both the pre- and post-surveys;
- Individuals who were unable to read and understand the survey independently.

### 2.2. Study Instrument

A structured survey instrument was developed to evaluate participants’ knowledge and awareness of key infectious disease topics, specifically within the domains of microbiology and parasitology. The content of the survey was based on public health priorities and disease prevalence data among the indigenous Semai community, focusing on five thematic areas: (1) transmission of head lice, (2) intestinal worm infection, (3) importance of hand washing, (4) transmission of tuberculosis, and (5) infection via contaminated objects. These topics were selected in consultation with medical parasitologists, microbiologists, and public health experts to ensure cultural and contextual relevance.

The survey consisted of five close-ended questions, each with three response options: “True”, “False”, and “Not sure”. These options allowed for the capture of uncertain knowledge while minimizing guesswork. A correct answer was awarded a score of 1, while both incorrect and “Not sure” responses were scored as 0, resulting in a maximum total score of 5 per respondent. This scoring method has been used in similar health literacy and education interventions.

The questionnaire was reviewed and pre-tested by three groups of content experts—medical parasitologists, microbiologists, and public health—for content validity, cultural appropriateness, and clarity. Although no formal psychometric validation was conducted due to the small and specific population, a pilot test was carried out with a small subset (*n* = 10) of the community to ensure the clarity and appropriateness of the items. Items were translated into Bahasa Malaysia and back-translated to confirm accuracy and semantic consistency. Feedback from the expert panel and pilot testing led to minor rewording for improved simplicity and comprehension. To reduce measurement bias, all questionnaires were administered in Bahasa Malaysia by trained researchers. The survey was conducted in a one-on-one setting to facilitate understanding without influencing participant responses. Participant identifiers (age and name) were collected to match pre- and post-test forms but were anonymized during analysis to maintain confidentiality. Given the quiz’s brevity and categorical scoring format, calculating Cronbach’s alpha would not have provided a meaningful assessment of internal consistency. The quiz was pilot-tested for face and content validity with subject matter experts as well as checked for clarity and cultural appropriateness.

### 2.3. Intervention

A health education initiative was provided at multiple interactive stations, each designed to deliver specific content on the prevention of infectious diseases using experiential learning techniques. The intervention was focused on five core thematic topics, selected based on universal infectious disease concerns and knowledge gaps in the community:

(1) Hand hygiene—to demonstrate correct hand washing and its significance in preventing the transmission of germs;

(2) Disease transmission awareness—to show how infection is transmitted by unhygienic surfaces and how lack of hygiene can spread infection, with an explanation of transmission pathways of pathogens;

(3) Head lice—to dispel common myths (e.g., that they fly) and describe appropriate treatment and prevention measures;

(4) Intestinal worm infections—to inform about the severity of parasitic infections and promote hygienic practices to prevent reinfection, especially by unclean hands or contaminated food;

(5) Tuberculosis (TB)—to help explain TB transmission, symptoms, and prevention, dispelling key myths such as believing TB is not an airborne infection.

Instruction methods included visual demonstrations, hands-on exercises, participatory games, and guided exhibitions. Everything was explained in simple, locally spoken Bahasa Malaysia with local suitable illustrations to achieve high participation and learning retention.

Each station was facilitated by trained facilitators who provided feedback and corrected misinformation in real-time as participants engaged. This structured, theme-based, and culturally appropriate approach was designed to increase disease knowledge, correct harmful beliefs, and provide long-term behavior change in prevention of infectious diseases.

### 2.4. Data Collection

The survey was distributed in hard copy, with invitations handed out to participants during the community health education program. A pre-survey was administered and collected before participants entered the program area. The community health education program then served as the central intervention. Upon completion of the educational sessions, participants were provided with the post-survey, which contained the same set of questions, and the form were collected by the researcher. Each participant completed the survey individually, without any external influence, to ensure the accuracy of the data. Each respondent was assigned an identification (ID) number, and participants’ identifying information was not recorded to ensure confidentiality.

### 2.5. Statistical Analysis

Data were manually entered into Microsoft Excel and then exported for analysis using the Statistical Package for Social Science (SPSS) version 30.0 (IBM Corp., Armonk, NY, USA). Correct answers were scored 1, with incorrect and “Not sure” being scored 0, with a total survey score of 5. Higher scores reflected greater knowledge and awareness.

Descriptive statistics were utilized to summarize data, with frequencies and percentages reported for categorical variables, and means and standard deviation are reported for continuous variables. Kolmogorov–Smirnov and Shapiro–Wilk tests were conducted to test for normality. Since the data were not normally distributed, the Wilcoxon signed-rank test was utilized for the comparison of pre-test and post-test means scores. Statistical significance was established at a *p*-value < 0.05, with the 95% confidence interval (CI) reported.

Although participant age was documented, no other demographic or behavioral controls were statistically controlled for in the analysis. Because each subject served as their own control in the within-subject pre-test-post-test design, inter-individual variability was minimized. However, age, gender, education level, and prior exposure to health education were not controlled because of the limited sample size. Future studies would need to account for the adjustment for these putative confounders to maximize the findings’ strength and subgroup-specific interpretation.

## 3. Results

### 3.1. Age Distribution

**Figure 1:**
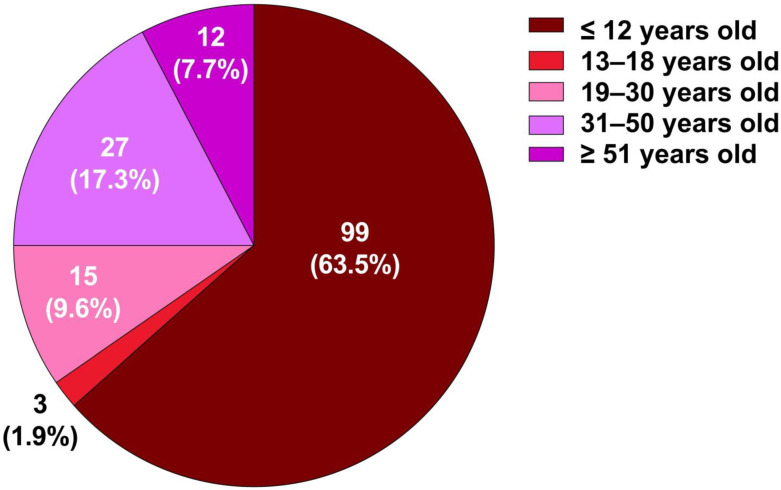
Age distribution of survey participants.

A total of 156 participants were from the Semai indigenous subgroup in Perak, Malaysia, and we provide detailed age distribution (7–69 years) analysis in Supplementary Table S1, with a median age of 12.0 years (interquartile range: 10–31 years), indicating a predominantly younger population. The age distribution ranged from 8 to 69 years and was heavily skewed towards children aged 12 years and below, who comprised 63.5% of the total respondents. This suggested that the health education program was either targeted toward younger individuals or conducted in an environment where children were the primary demographic, such as a school, community health program, or family-based setting. In contrast, the adolescents (13–18 years) constituted only 1.9%, the lowest proportion among all age groups, indicating lower engagement or representation. The adult groups (19–30, 31–50, and ≥51 years) collectively accounted for 34.6% of the participants, with the 31–50 years category representing the second-largest group (17.3%), followed by 19–30 years (9.6%) and ≥51+ years (7.7%) (Figure 1). Although the intervention was designed for the broader community, including adults and adolescents, the majority of participants were children due to the school-based setting and their greater availability during the program period. This unintentional bias was acknowledged and discussed as a limitation.

### 3.2. Pre-Test and Post-Test Scores Across Different Age Groups

**Figure 2:**
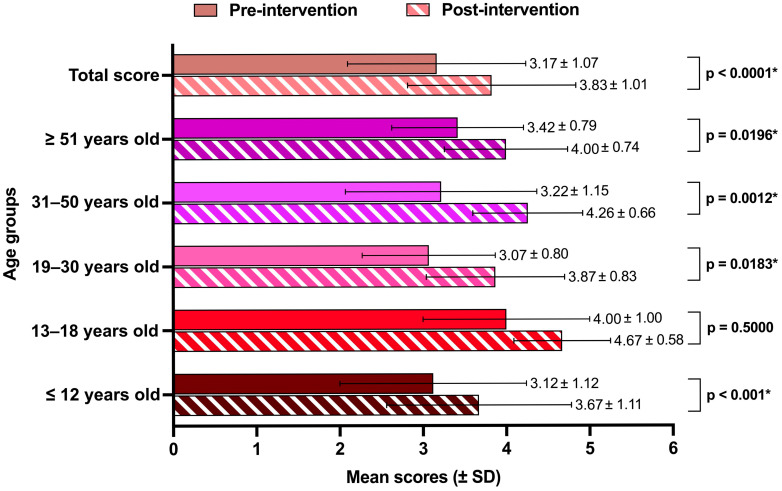
Pre- and post-test mean scores (±SD) across different age groups. * *p*-value < 0.05.

The pre- and post-survey mean scores across all age groups are summarized in Figure 2. Overall, a substantial improvement in knowledge was observed (*p* < 0.0001) with mean pre-survey scores (3.17 ± 1.07) rising in the post-survey period (3.83 ± 1.01), 95% CI: −0.86 to −0.46. These findings indicate that the community health intervention effectively enhanced knowledge and awareness among participants.

When analyzed by age group, statistically significant improvements were observed across all groups except adolescents aged 13–18 years (*p* > 0.05), possibly due to the small size of this group. The baseline knowledge varied across age groups, with mean pre-survey scores ranging from 3.07 to 3.42, except for adolescents (13–18 years), who exhibited a relatively higher baseline awareness (4.00 ± 1.00). The small representation of adolescents may have affected the statistical power of the analysis and suggested a potential ceiling effect, limiting the measurable impact of the intervention.

The most substantial improvement was observed among middle-aged adults, with mean difference of 1.04 ± 1.48, followed by young adults (0.80 ± 1.08), suggesting their strong receptivity to health-related information. Adolescents (13–18 years) exhibited a moderate improvement (0.67 ± 1.15), while children (≤12 years) and older adults showed the least improvement (0.55 ± 1.31 and 0.58 ± 0.67, respectively), potentially due to entrenched misconceptions or lower adaptability to new information. These results indicate the need for targeted, age-specific health education approaches to address knowledge gaps effectively.

### 3.3. Variations in Health Awareness Knowledge by Age Group

Statistically significant improvements were observed in several knowledge domains following the intervention. The greatest gain was in handwashing techniques (mean difference = 0.41 ± 0.55, *p* < 0.001), with significant improvements across all age groups except adolescents (13–18 years), where the change was not statistically significant. This highlights the intervention’s effectiveness in promoting hygiene awareness, particularly among children and adults.

Moderate but significant improvements were also noted in knowledge related to tuberculosis and germ transmission (*p* = 0.0022 and *p* = 0.0015, respectively). Young adults (19–30 years) showed significant gains in TB knowledge (*p* = 0.0455), while children and middle-aged adults improved in understanding germ transmission (*p* = 0.0389 and *p* = 0.0455, respectively).

**Figure 3:**
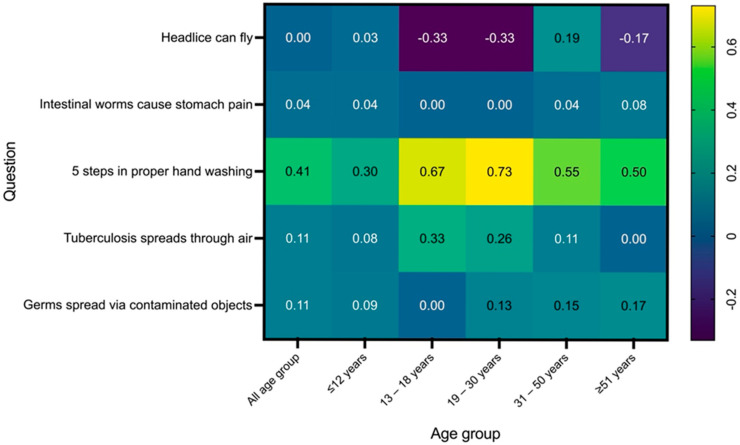
Mean difference in pre- and post-test scores by question and age group.

In contrast, knowledge about intestinal worms and head lice showed minimal improvement. The mean difference for intestinal worms was only 0.04 (*p* = 0.1091), and no significant changes were observed for head lice across age groups (*p* > 0.05), indicating persistent misconceptions. These findings suggest a need for more targeted educational strategies to address the deeply rooted myths in these areas (Table 1 and Figure 3).

## 4. Discussion

Health awareness is essential for disease prevention. However, many communities continue to face misconceptions and gaps in knowledge [13,14,15,16]. This study offers new insights into hygiene practices and health awareness, contributing to the limited data on community knowledge gaps and the effectiveness of educational interventions.

Rather than focusing on one disease, this study examined a variety of common health topics. These included tuberculosis, head lice, intestinal worms, gastrointestinal illnesses, and hygiene behaviors like handwashing. Although these topics are clinically different, they all relate to public health and are often misunderstood. By addressing them together, we aimed to assess health literacy and identify priority areas for education.

Children aged 8–12 years formed the majority (63.5%) of the participants, reflecting the survey’s school and community-based settings. Health education programs are often targeted at this age group to encourage the early adoption of hygiene practices [17]. In contrast, adolescents made up only 1.9% of the participants, raising concerns about their limited engagement, likely due to outreach gaps, competing priorities, or lack of interest in conventional methods. Given their digital affinity, strategies such as gamified apps, mobile learning platforms, and social-media-based campaigns have shown promise and may enhance future reach and engagement [18].

Adults accounted for 34.6% of participants. The largest subgroup was aged 31–50 years (17.3%), followed by those 19–30 years (9.6%) and those over 51 years (7.7%). Adult participation suggests family and community involvement, which is important as parents often influence children’s health habits [19]. However, the lower involvement of older adults and adolescents indicates a need for tailored outreach. Barriers for older adults may include limited access, cognitive changes, or adherence to traditional beliefs. Age-specific strategies are needed to address these issues and promote inclusive health education [20,21,22].

The pre- and post-survey assessments showed improved health awareness across all age groups. Adults aged 31–50 years demonstrated the most significant gains. Adolescents (13–18 years) showed a numerical improvement (0.67 ± 1.15), but this was not statistically significant, likely due to their small sample size (only 1.9% of the participants) and high baseline scores. While their absolute gains were notable, they cannot be confidently interpreted as meaningful due to the low statistical power. Nevertheless, the cognitive readiness of adolescents and their potential responsiveness to interactive or digital interventions make them a crucial target group for future health education efforts [23,24,25,26]. Adolescents tend to favor digital or interactive approaches, while adults often engage more with health information that influences family well-being [26]. In contrast, older adults (51+ years) showed the least improvement. This may have resulted from ingrained misconceptions, limited digital literacy, and resistance to change. These findings support the need for demographic-specific education methods. Peer discussions, workplace seminars, and game-based learning may improve effectiveness across different age groups [27,28,29].

In order to achieve the maximum interpretive value of our findings, our results may be explained with the aid of the Health Belief Model (HBM). For example, heightened awareness can be correlated with heightened perceptions of disease susceptibility and severity among adolescents and adults, who responded more strongly [30]. The HBM also supports the use of tailored messaging to influence perceived benefits and reduce perceived barriers, which was notable particularly among older adults whose participation was lower [31]. In addition, aspects that highlight cultural competence were incorporated into the intervention through the use of shared examples, native facilitators, and culturally appropriate materials [32]. These strategies are in accord with the principles of cultural competence, which ensure respect for traditional beliefs while scientifically sound health messages are disseminated. Utilizing such frameworks helps explain the differential knowledge uptake between age groups and informs the refinement of future programs.

Despite the knowledge gains, persistent misconceptions were identified. Alarmingly, 92.07% believed tuberculosis is not airborne. This indicates a serious lack of understanding of disease transmission [33,34,35]. The misconception may stem from the limited health education or cultural beliefs that misattribute the disease to sanitation or genetics. Similarly, 81.71% believed handwashing was unnecessary if hands appeared clean, and 77.44% thought water alone was sufficient. These beliefs ignore that pathogens cannot be seen, which may lead to unsafe hygiene practices [36,37].

There were also myths about parasitic infections [38]. About 89.63% believed intestinal worms only affected children, and 40.24% thought head lice could fly. The former may have resulted from child-focused deworming campaigns, while the latter likely stemmed from confusion with other insects. These errors can hinder proper prevention and treatment. Correcting such misconceptions requires structured education campaigns, especially in schools, community programs, and digital platforms, where misinformation spreads easily [37,38,39,40].

Health awareness scores varied with age. Children and adolescents had the lowest scores, 55 and 45 out of 100, respectively. Children often rely on fragmented school or parental education. Teenagers may resist traditional learning and prefer peer or online content [39]. In contrast, middle-aged adults (36–55 years) had the highest scores, likely due to caregiving roles and more exposure to health programs [41,42]. Older adults had lower scores than middle-aged adults, possibly due to outdated knowledge or limited exposure to new health information. Age-related cognitive changes may also have played a role. For this group, simple and repeated messaging may be more effective [43].

As per the evidence and gaps, post-intervention control has to prioritize confirmatory follow-up tests and resolving he remaining misconceptions. The short-term gains have to be followed by long-term consolidation via community-based or digital education, as relevant according to age group, with an emphasis on adolescents and older adults [44]. On a practical level, incorporating community health volunteers and educators for repeat sessions may prove to be helpful in reinforcing correct information and fostering trust. Peer outreach and culturally relevant materials can also facilitate participation. While this study focused on knowledge gains, practical impacts of such programs can be achieved by integrating behavior change communication, increasing the availability of hygiene facilities, and encouraging participation at the family level. As a step forward, cheap, locally available options like ginger or garlic extracts can be explored for antimicrobial education in resource-poor settings, not only as teaching aids but as traditional health promotion [45]. Ultimately, a multilayered strategy combining clinical acumen, cultural humility, digital technology, and community ownership is required for sustained health literacy and infectious disease control.

Our findings align with broader evidence from indigenous health interventions globally, where culturally adapted, community-led, and participatory education strategies have shown success. Similar to reports from Aboriginal Australian and Māori communities, interventions that respect traditional beliefs and use local languages improve engagement and outcomes [46,47]. For instance, in Aboriginal populations, health literacy improved significantly when programs incorporated visual aids, storytelling, and community leaders [48]. Among the Māori, embedding health education within cultural frameworks such as whānau ora enhanced both participation and the retention of knowledge [46]. Inuit health programs also stress the importance of community involvement and culturally relevant content [49]. These parallels underscore the importance of tailoring health interventions to indigenous worldviews, reinforcing the relevance and transferability of our approach to broader indigenous settings.

This research has several limitations. The use of convenience sampling may have led to selection bias, limiting the generalizability of the findings to the larger Semai indigenous population. This study’s sample was predominantly composed of children aged 12 and below, who comprised 63.5% of the sample. While this is reflective of the accessibility and availability of this age group to community-based intervention, it poses a potential sampling bias that does not allow for generalizability to all ages. The representation of adults and older adults was comparatively lower, potentially skewing total gains in knowledge and concealing age-specific issues in health awareness [50]. Secondly, education data were not collected within the context of this study, thus narrowing our ability to examine the relationship between health knowledge and the degree of formal schooling. This is a significant limitation, particularly given that previous studies have identified low health literacy and low formal education as the principal barriers to effective health interventions amongst indigenous populations, like the Orang Asli in Malaysia [2,8]. Future studies ought to include a more representative age allocation and consider education levels as variables in order to more accurately capture their impact on health knowledge and intervention outcomes. The reliance on self-reported data introduced potential social desirability bias and the risk of misunderstanding survey questions [51]. Furthermore, this study assessed only short-term changes in knowledge immediately post-intervention and did not evaluate long-term retention or actual behavior change, such as improved hygiene practices or health-seeking behaviors [52]. Longitudinal designs with behavioral measures such as hygiene practices or the use of health services should be employed in future studies to further evaluate the long-term impact of the intervention; also, focus groups are essential to understand sustained knowledge retention, behavior change, and the maintenance of community beliefs [53,54].

## 5. Conclusions and Future Perspectives

This study provides valuable information on the health awareness and widespread misconceptions of the Semai indigenous people, and certain educational interventions are essential. While general awareness levels were moderate, some significant gaps existed, particularly in the knowledge of disease spread of diseases such as tuberculosis, the importance of proper hand washing, and misconceptions regarding parasitic infections such as head lice. These findings underscore the importance of culturally relevant, community-level health education programs that involve local leaders and employ interactive learning strategies such as demonstrations, role-playing, and visual aids in indigenous languages to maximize knowledge retention and relevance. Health education integrated in school curricula can promote long-term behavior change, while regular community health check-ups and sensitization sessions can promote hygiene behavior. Additionally, making use of online media through short educational clips, infographics, and social media campaigns is a great way to reach out to youth, who are increasingly relying on online sources for information. In the future, longitudinal studies are recommended to determine the long-term impact of such interventions on indigenous people. In addition, the development of new, transferable, educational methods must be established in order to allow for ongoing improvements in health literacy and for the prevention of disease in indigenous peoples.

## Figures and Tables

**Table 1:** Mean pre- and post-test differences in score (±SD) for each question in across age groups. * *p*-value < 0.05.

Question	Age Group	Pre-Test Mean ± SD	95% CI	Post-Test Mean ± SD	95% CI	Mean Difference (Post–Pre) ± SD	*p*-Value
Question 1: Head lice can fly	All age group	0.58 ± 0.50	0.50–0.66	0.58 ± 0.50	0.50–0.66	0.00 ± 0.60	1.0000
	≤12 years	0.63 ± 0.49	0.53–0.73	0.66 ± 0.48	0.56–0.76	0.03 ± 0.69	0.6015
	13–18 years	1.00 ± 0.00	-	0.67 ± 0.58	−0.77–2.11	−0.33 ± 0.58	0.3173
	19–30 years	0.60 ± 0.51	0.32–0.88	0.27 ± 0.46	0.02–0.52	−0.33 ± 0.69	0.0588
	31–50 years	0.37 ± 0.49	0.18–0.56	0.56 ± 0.51	0.36–0.76	0.19 ± 0.71	0.1655
	≥51 years	0.50 ± 0.52	0.17–0.83	0.33 ± 0.49	0.02–0.64	−0.17 ± 0.71	0.1573
Question 2: Intestinal worms cause stomach pain	All age group	0.91 ± 0.29	0.86–0.95	0.95 ± 0.23	0.91–0.98	0.04 ± 0.31	0.1091
	≤12 years	0.88 ± 0.33	0.81–0.95	0.92 ± 0.27	0.87–0.97	0.04 ± 0.43	0.2482
	13–18 years	1.00 ± 0.00	-	1.00 ± 0.00	-	0.00 ± 0.00	N/A
	19–30 years	1.00 ± 0.00	-	1.00 ± 0.00	-	0.00 ± 0.00	N/A
	31–50 years	0.96 ± 0.19	0.88–1.04	1.00 ± 0.00	-	0.04 ± 0.19	0.3173
	≥51 years	0.92 ± 0.29	0.74–1.10	1.00 ± 0.00	-	0.08 ± 0.29	0.3173
Question 3: 5 steps in proper hand washing	All age group	0.14 ± 0.35	0.09–0.20	0.55 ± 0.50	0.47–0.63	0.41 ± 0.55	<0.0001 *
	≤12 years	0.14 ± 0.35	0.07–0.21	0.44 ± 0.50	0.34–0.54	0.30 ± 0.61	<0.0001 *
	13–18 years	0.33 ± 0.58	−1.11–1.77	1.00 ± 0.00	-	0.67 ± 0.58	0.1573
	19–30 years	0.00 ± 0.00	-	0.73 ± 0.46	0.48–0.98	0.73 ± 0.46	0.0009 *
	31–50 years	0.19 ± 0.40	0.03–0.35	0.74 ± 0.45	0.56–0.92	0.55 ± 0.60	0.00027 *
	≥51 years	0.17 ± 0.39	−0.08–0.42	0.67 ± 0.49	0.36–0.98	0.50 ± 0.63	0.0143 *
Question 4: Tuberculosis spreads through air	All age group	0.75 ± 0.43	0.68–0.82	0.86 ± 0.35	0.80–0.91	0.11 ± 0.42	0.0022 *
	≤12 years	0.71 ± 0.46	0.62–0.80	0.79 ± 0.41	0.71–0.87	0.08 ± 0.62	0.0881
	13–18 years	0.67 ± 0.58	−0.77–2.11	1.00 ± 0.00	-	0.33 ± 0.58	0.4226
	19–30 years	0.67 ± 0.49	0.40–0.94	0.93 ± 0.26	0.79–1.07	0.26 ± 0.55	0.0455 *
	31–50 years	0.85 ± 0.36	0.71–0.99	0.96 ± 0.19	0.88–1.04	0.11 ± 0.41	0.1797
	≥51 years	1.00 ± 0.00	-	1.00 ± 0.00	-	0.00 ± 0.00	N/A
Question 5: Germs spread via contaminated objects	All age group	0.79 ± 0.41	0.72–0.86	0.90 ± 0.30	0.85–0.95	0.11 ± 0.41	0.0015 *
	≤12 years	0.77 ± 0.42	0.69–0.85	0.86 ± 0.35	0.79–0.93	0.09 ± 0.55	0.0389 *
	13–18 years	1.00 ± 0.00	-	1.00 ± 0.00	-	0.00 ± 0.00	N/A
	19–30 years	0.80 ± 0.41	0.57–1.03	0.93 ± 0.26	0.79–1.07	0.13 ± 0.49	0.1573
	31–50 years	0.85 ± 0.36	0.71–0.99	1.00 ± 0.00	-	0.15 ± 0.36	0.0455 *
	≥51 years	0.83 ± 0.39	0.58–1.08	1.00 ± 0.00	-	0.17 ± 0.39	0.1573

## Data Availability

The data will be made available upon request.

## Supplementary Materials

The following supporting information can be downloaded at: https://www.mdpi.com/article/10.3390/tropicalmed10070191/s1, Supplementary Table S1. Mean differences in survey score across all age groups.
