# Supplementary material for: Impact of Health Education on Infectious Disease Knowledge in Indigenous Communities in Northwestern Malaysia
Source: Trop Med Infect Dis. 2025 Jul 9;10(7):191. doi: 10.3390/tropicalmed10070191 (PMC12297879; doi:10.3390/tropicalmed10070191)
Supplement: Supplementary file 1 [file tropicalmed-10-00191-s001.zip › tropicalmed-3660313-supplementary.pdf]

Supplementary Table S1. Mean differences in survey score across all age groups. *p-value <0.05.

Age group	Pre-test Mean ± SD	95% CI	Post-test Mean ± SD	95% CI	Mean difference (Post–Pre) ± SD	p-value
**All age group**	3.17 ± 1.07	3.00 – 3.34	3.83 ± 1.01	3.67 – 3.99	0.66 ±1.29	**<0.0001***
**≤12 years**	3.12 ± 1.12	2.90 – 3.34	3.67 ± 1.11	3.45 – 3.89	0.55 ± 1.31	**< 0.0001***
**13 – 18 years**	4.00 ± 1.00	1.51 – 6.49	4.67 ± 0.58	3.23 – 6.11	0.67 ± 1.15	0.5000
**19 – 30 years**	3.07 ± 0.80	2.62 – 3.51	3.87 ± 0.83	3.40 – 4.33	0.80 ± 1.08	**0.0183***
**31 – 50 years**	3.22 ± 1.15	2.77 – 3.68	4.26 ± 0.66	4.00 – 4.52	1.04 ± 1.48	**0.0012***
**≥51 years**	3.42 ± 0.79	2.91 – 3.92	4.00 ± 0.74	3.53 – 4.47	0.58 ± 0.67	**0.0196***
